# Clinical efficacy and safety of apatinib combined with S-1 in advanced esophageal squamous cell carcinoma

**DOI:** 10.1007/s10637-019-00866-5

**Published:** 2019-10-24

**Authors:** Jian Zhao, Junmei Lei, Junyan Yu, Chengyan Zhang, Xuefeng Song, Ninggang Zhang, Yusheng Wang, Suxiang Zhang

**Affiliations:** 1 Shanxi Medical University, Taiyuan, Shanxi 030001 People’s Republic of China; 2 Department of Digestive, Affiliated Cancer Hospital of Shanxi Medical University, Taiyuan, Shanxi 030013 People’s Republic of China; 3 Department of Oncology, Jincheng General Hospital, Jincheng, Shanxi 048000 People’s Republic of China; 4 Department of Oncology, Affiliated Peace Hospital of Changzhi Medical College, Changzhi, Shanxi 046000 People’s Republic of China; 5 Capital Medical University, Beijing, 100069 People’s Republic of China

**Keywords:** Esophageal squamous cell carcinoma, Apatinib, S-1, Adverse effect, Survival

## Abstract

*Background* Esophageal cancer is a very common malignant tumor in China, especially esophageal squamous cell carcinoma (ESCC), but there is currently no effective treatment for patients after first-line chemotherapy failure. Apatinib has shown promising outcomes in treatment with various solid tumors. *Objectives* To evaluate the clinical efficacy and safety of apatinib combined with S-1 in the treatment of advanced ESCC patients after first-line chemotherapy failure. *Methods* In this prospective study, fifteen patients with advanced ESCC who failed first-line chemotherapy were enrolled from Nov 2016 to Apr 2019. Patients received the combination therapy with apatinib (250-500 mg, once daily) plus S-1 (40–60 mg based on body surface area, twice daily). Primary endpoint was progression-free survival (PFS). Secondary endpoints included overall survival (OS), disease control rate (DCR) and objective response rate (ORR). Adverse events (AEs) were recorded to evaluate the safety. *Results* A total of 12 patients were included in the efficacy analysis. The median PFS was 6.23 months, and the median OS was 8.83 months. Two (16.67%) patients achieved partial remission, 9 patients (75.00%) achieved stable disease and 1 (8.33%) patient achieved progressive disease. DCR and ORR was 91.67%and 16.67%, respectively. Most frequent AEs were hypertension, myelosuppression, weakness, hemorrhage, hand-foot syndrome, total bilirubin elevation, sick, proteinuria, oral ulcer, loss of appetite, and transaminase elevation. The most AEs were in grade I~II. *Conclusion* The combination therapy of apatinib plus S-1 was effective and well tolerated in the treatment of advanced ESCC patients after first-line chemotherapy failure. The combination therapy has the potential to be a potent therapeutic option for advanced ESCC patients after first-line chemotherapy failure.

## Introduction

Esophageal cancer is the sixth leading cause of cancer death worldwide [1]. It is also a common malignant tumor in China, especially esophageal squamous cell carcinoma (ESCC), with obvious regional distribution characteristics [2, 3]. However, most patients were diagnosed at the advanced stage, resulting in the loss of chance to receive the surgery [4]. For early esophageal cancer, endoscopic submucosal dissection (ESD) and endoscopic mucosal resection (EMR) may be preferred [5]. For advanced patients, local radiotherapy and/or systemic chemotherapy are required. Chemotherapy can alleviate clinical symptoms, improve quality of life, and prolong survival. Currently, first-line chemotherapy for advanced esophageal cancer mainly includes fluorouracil-based or platinum-containing regimens. However, there is no effective treatment for patients after at least first-line chemotherapy failure. Therefore, the novel therapeutic regimen for advanced ESCC is urgently needed.

In recent years, vascular endothelial growth factor receptor (VEGFR) has been shown to be an important anti-cancer target in the targeted therapy of solid tumors. Since 1970s, Folkman et al. has provided evidence that the growth and metastasis of solid tumors are associated with angiogenesis [6–8]. Bevacizumab is the first drug that approved by US Food and Drug Administration to inhibit tumor angiogenesis. It is actually a humanized variant of anti-VEGF antibody that specifically binds to VEGF-A to promote tumor vascular normalization [9]. VEGF exerts angiogenesis effect by binding to various transmembrane proteins and there are three primary receptors, including VEGFR-1, VEGFR-2, and VEGFR-3. Moreover, several studies have shown that ESCC growth can be inhibited by selectively inhibiting VEGFR-1 and VEGFR-2 [10–12].

Apatinib is an oral small-molecule tyrosine kinase inhibitor (TKI) that selectively binds to and inhibits VEGFR2. A retrospective study suggested that apatinib may be beneficial for patients with ESCC, the objective response rate (ORR) and disease control rate (DCR) were 24.2% and 74.2%, respectively [13]. S-1 is a fourth-generation, novel, orally active fluorouracil formulation, consisting of tegafur (FT; a prodrug of 5-FU), 5-chloro-2,4-dihydroxypyridine (CDHP) and potassium oxonate (Oxo), in a molar ratio of 1:0.4:1 [14]. CDHP can maintain prolonged efficacious 5-FU concentrations in the blood by inhibiting dihydropyrimidine dehydrogenase (DPD). Oxo can suppress the gastrointestinal toxicity of 5-FU and without affecting the antitumor activity of 5-FU [15]. At present, there is no relevant research. Our objective is to preliminarily evaluate the efficacy and safety of combination drug therapy.

Herein we present an interim analysis of a prospective study to evaluate the efficacy and safety of apatinib combined with S-1 in advanced ESCC.

## Materials and methods

### Patients and study design

This prospective study enrolled 15 patients with advanced ESCC who experienced at least one failure of first-line chemotherapy from Nov 2016 to Apr 2019 at Affiliated Cancer Hospital of Shanxi Medical University. The study was conducted in accordance with the Declaration of Helsinki and approved by the Ethics Committee of Affiliated Cancer Hospital of Shanxi Medical University. Written informed consent was obtained from each subject. The study was registered in Chinese Clinical Trials. gov. (ChiCTR-OIH-17012822).

Inclusion criteria were as follows: (1) patients were diagnosed with stage IV ESCC by histopathology and/or cytology; (2) had failed first-line chemotherapy; (3) presence of objectively measurable tumor lesions; (4) had an Eastern Cooperative Oncology Group (ECOG) physical status score of 0–2; (5) without the obvious abnormalities in heart, liver and kidney function, and without the risk of bleeding and thrombosis, and chemotherapy contraindications.

### Drug administration

Apatinib was administered at an initial dose of 250 mg once daily. If the initial dose was well tolerated after one week, the dose of apatinib was adjusted to 500 mg, once daily. S-1 was administered a dose of 40–60 mg twice daily for 4 weeks followed by a 2 weeks drug interruption. Each treatment cycle was 6 weeks. The dose of S-1 was determined based on body surface area (BSA): 40 mg (BSA < 1.25 m^2^), 50 mg (BSA 1.25–1.5 m^2^), and 60 mg (BSA ≥1.5 m^2^). Apatinib and S-1 were provided by Jiangsu HengRui Medicine Co., Ltd. (Lianyungang, Jiangsu, China).

### Efficacy and safety evaluation

Tumor response was assessed every 6 weeks by using computed tomography (CT) and tumor markers. The primary efficacy endpoint was progression-free survival (PFS).

Tumor response was categorized as complete response (CR), partial response (PR), stable disease (SD), and progressive disease (PD) according to Response Evaluation Criteria in Solid Tumors version 1.1(RECIST v1.1) [16]. The secondary efficacy endpoints were disease control rate (DCR), objective response rate (ORR) and over survival (OS). DCR was defined as the number of patients with CR, PR, and SD among all patients. ORR was defined as the number of patients with the best tumor response (CR and PR) among all patients. Adverse events (AEs) were recorded to evaluate the safety. All AEs were graded according to the Common Terminology Criteria for Adverse Events (CTCAE) Version 4.0, ranging from 0 to 4.

### Statistical analysis

PFS data for patients without disease progression or loss of follow-up were censored at the time of last tumor assessment. OS data for patients who survived or lost follow-up were censored at the time of last confirmed exposure. Safety assessment was analyzed in safety analysis set (SAS), which included all patients who had received at least one dose of study treatment. SPSS version 19.0 (SPSS Inc. USA) was used for all statistical analyses. Quantitative data were described using mean, standard deviation (SD), median, and interquartile range, while qualitative data were described by number or percentage. OS and PFS were estimated by using the Kaplan-Meier method. A value of *p* < 0.05 was considered as statistically significant.

## Results

### Patients baseline

A total of 15 patients were enrolled in present study (Table 1). All patients have experienced at least one failure of first-line chemotherapy. The present study enrolled 8 (53.3%) males and 7 (46.7%) females, with the median age of 68 (range, 57–76). The majority of ESCC are located in the lower esophagus and the majority pathological subtype is poorly differentiated squamous cell carcinomas. All patients had an ECOG (Eastern Cooperative Oncology Group) performance status score of 0 or 2.

**Table 1:** Patients characteristics

	N = 15	
	NO	%
Age (years)		
Median	68 50–76	
Range		
Gender		
Male	8	53.3%
Female	7	46.7%
Location		
Cervical esophagus	1	6.7%
Upper thoracic	2	13.3%
Middle thoracic	4	26.7%
Lower thoracic	6	40.0%
Esophagogastric junction	0	0.0%
NA	2	13.3%
Surgery		
Yes	6	40.0%
No	9	60.0%
Differentiation		
Poorly differentiated	7	46.7%
Moderately or poorly differentiated	2	13.3%
Moderately differentiated	3	20.0%
Middle to well differentiated	1	6.7%
Well-differentiated	0	0%
NA	2	13.3%
ECOG score		
1	8	53.3%
2	7	46.7%
The numbers of metastases		
≤2	6	40.0%
>2	6	40.0%
NA	3	20.0%
Previous treatment		
First-line	1	6.7%
Second-line	5	33.3%
Third-line	4	26.7%
Fourth-line	1	6.7%
Fifth-line	2	13.3%
NA	2	13.3%

*ECOG*, Eastern Cooperative Oncology Group

### Efficacy

**Fig. 1:**
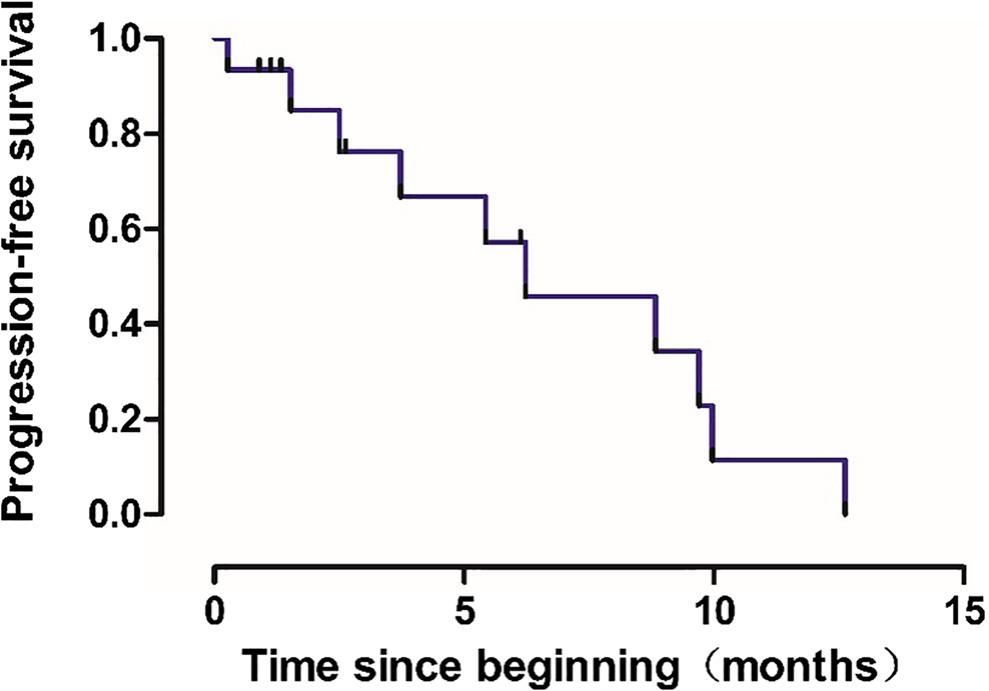
Kaplan-Meier analysis of progression-free survival in patients treated with apatinib combined with S-1 (*n* = 15). mPFS: 6.23 months (95% CI, 1.68–10.78)

**Fig. 2:**
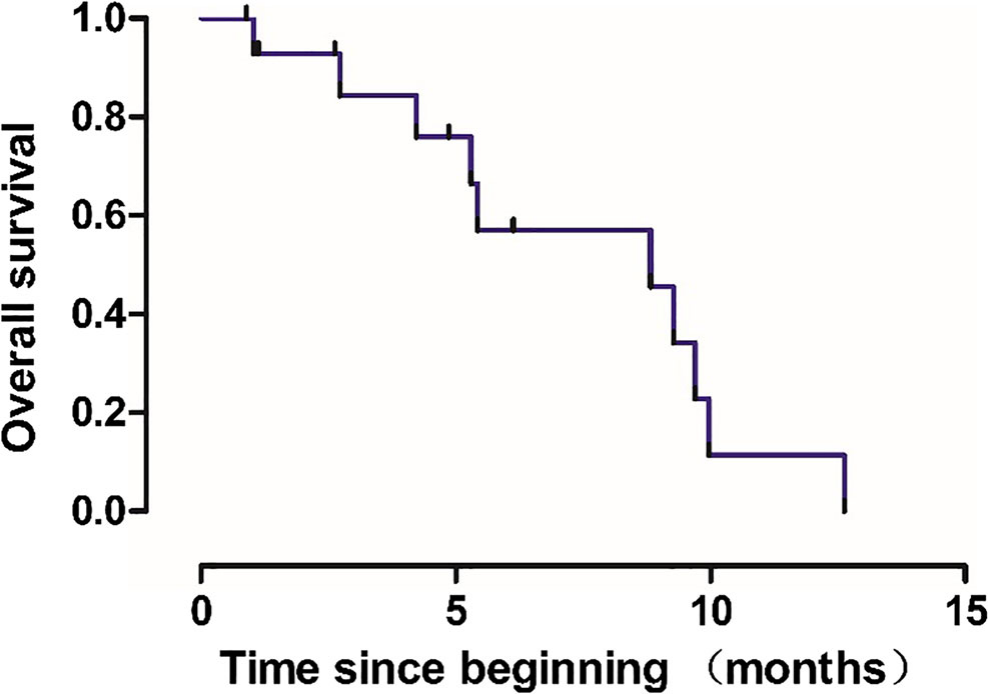
Kaplan-Meier analysis of overall survival in patients treated apatinib combined with S-1 (n = 15). mOS: 8.83 months (95% CI, 3.67–13.99)

Among 15 eligible patients, 12 patients were included in the efficacy analysis. The reason for patients not included in the efficacy analysis were insufficient medication time in 2 cases and withdrawal from the trial because of AEs in 1 case. Among the 12 patients, none of patients achieved CR, 2 (16.67%) patients achieved PR, 9 (75.00%) patients achieved SD and 1 (8.33%) patient achieved PD (Table 2). The DCR and ORR was 91.67%and 16.67%, respectively (Table 2). In all 15 patients, the median PFS was 6.23 months (95% CI, 1.68–10.78), and median OS was 8.83 months (95% CI, 3.67–13.99) (Figs. 1 and 2).

**Table 2:** Detailed information of patients in this trial

NO.	Enter the trail	Withdraw from the trail	The reason	Time of Death	Previous treatment	Dose	Optimal efficacy	PFS(month)	OS(month)
1	2016-11-09	2017-08-27	NA	2017-08-27	NA	NA	SD	9.70	9.70
2	2.17–03-23	2017-12-23	Death	2017-12-23	Fifth-line	500 mg	SD	8.83	8.83
3	2017-06-12	2018-04-07	Death	2018-04-07	Third-line	500 mg	PR	9.97	9.97
4	2017-10-11	2017-12-25	Progress	2018-01-01	Second-line	500 mg	PR	2.50	2.73
5	2017-10-15	2018-03-27	Death	2018-03-27	Third-line	250 mg	SD	5.43	5.43
6	2017-11-29	2017-12-07	Bleeding / perforation	2017-12-30	NA	250 mg	NA	0.27	1.03
7	2018-01-05	2018-07-08	NA	NA	Fifth-line	500 mg	SD	6.13	6.13
8	2018-01-17	2019–01–31	Death	2019–01–31	Third-line	500 mg	SD	12.63	12.63
9	2018-02-05	2018-04-25	NA	NA	Second-line	250 mg	SD	2.63	2.63
10	2018-04-11	2018-05-27	Poor compliance	2018-09-17	Second-line	NA	SD	1.53	5.30
11	2018-04-23	2018-08-13	Death	2018-08-28	Third-line	250 mg	SD	3.73	4.23
12	2018-06-22	2018-12-26	Anemia	2019-03-27	First-line	250 mg	SD	6.23	9.27
13	2018-12-05	2019-01-14	Progress	NA	Second-line	375 mg	PD	1.33	4.87
14	2019-03-27				Forth-line	250 mg	NA		
15	2019-04-03				Second-line	250 mg	NA		

*SD*, stable disease; *PD*, progressive disease; *PR*, partial response; *NA*, no answer; *PFS*, progression-free survival; *OS*, overall survival

### Safety

**Fig. 3:**
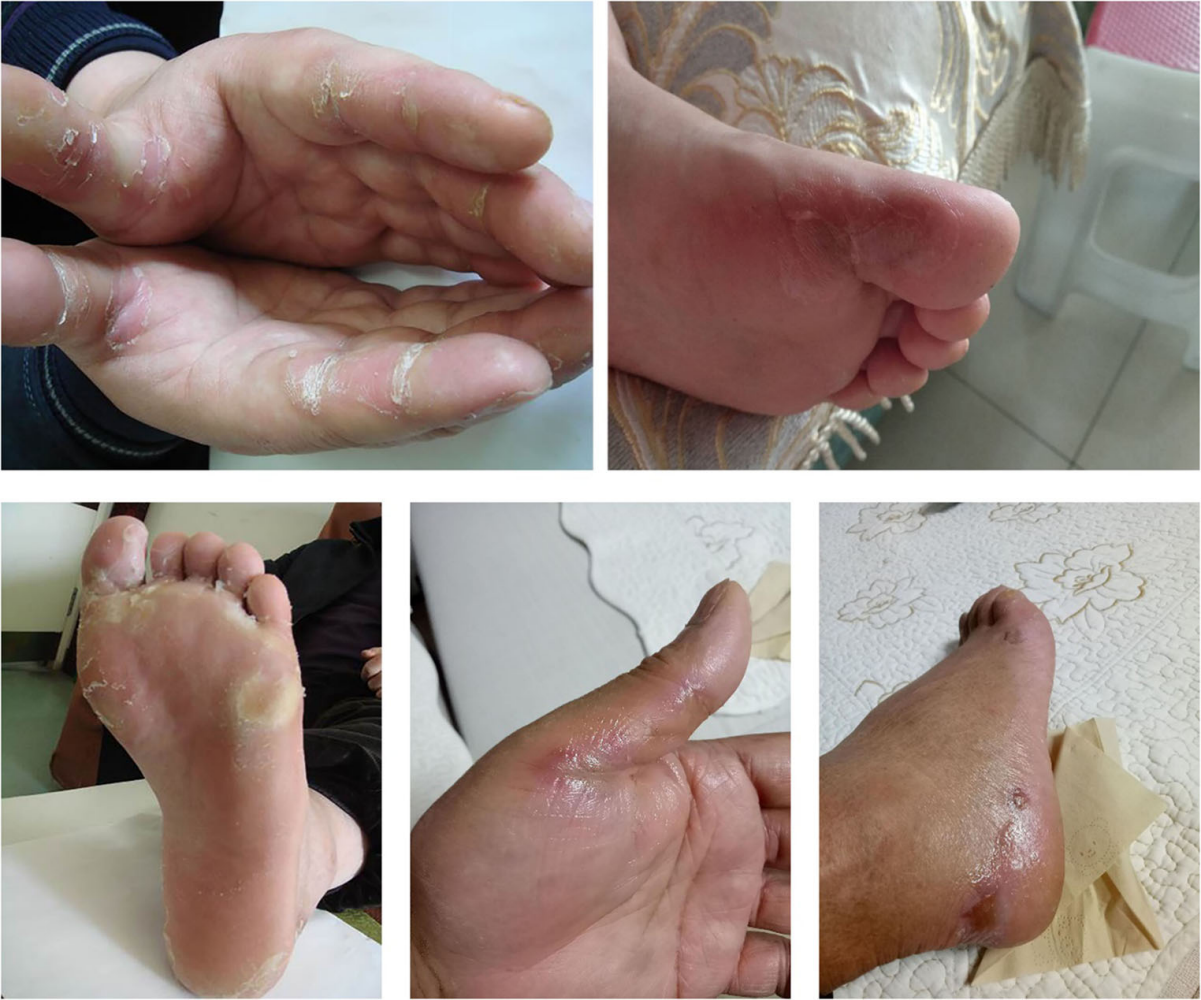
Hand-foot syndrome. These pictures were taken from three patients with hand-foot syndrome after apatinib combined with S-1, and they are tolerable after symptomatic treatment. (The consent and authorization have been obtained)

The main AEs were listed in Table 3. Most AEs were grade I and II, including hematotoxicity and non-hematologic toxicity. Most of hematotoxicity were thrombocytopenia (20.00%, 3/15) and leukocyte reduction (13.33%, 2/15); non-hematologic toxicity mainly manifested as hypertension (40.00%, 6/15), weakness (33.33%, 5/15), hemorrhage (20.00%, 3/15), hand-foot syndrome (HFS) (20.00%, 3/15), total bilirubin elevation (13.33%, 2/15), sick (13.33%, 2/15), proteinuria (6.67%, 1/15), oral ulcer (13.33%, 2/15), loss of appetite (6.67%, 1/15), and transaminase elevation (6.67%, 1/15). The main grade III AEs were HFS (6.67%, 1/15), proteinuria (6.67%, 1/15), thrombocytopenia (6.67%, 1/15) and hemorrhage (6.67%, 1/15). Besides, one patient experienced grade IV total bilirubin elevation (6.67%, 1/15) (Fig. 3).

**Table 3:** Adverse events

Adverse events	Grade				Incidence	Main grade 3–4 toxicities
	I	II	III	IV		
Hypertension	3	3			40.00%	0.00%
Thrombocytopenia	2		1		20.00%	6.67%
Weakness	2	3			33.33%	0.00%
Hemorrhage		2	1		20.00%	6.67%
Leukocyte reduction	1	1			13.33%	0.00%
Hand-foot syndrome		2	1		20.00%	6.67%
Total bilirubin elevation	1			1	13.33%	6.67%
Sick	2				13.33%	0.00%
Oral ulcer	2				13.33%	0.00%
Proteinuria			1		6.67%	6.67%
Loss of appetite	1				6.67%	0.00%
Transaminase elevation	1				6.67%	0.00%

## Discussion

This is an interim analysis of a single arm, single institutional, prospective, exploratory clinical study, the median PFS was 6.23 months, and the combination of apatinib and S-1 was found to have a manageable AEs. From the available data, the efficacy seems to be related to surgery, gender, apatinib’s dose, the numbers of metastases and previous treatment, but they are not statistically significant (Table 4).

**Table 4:** Univariate analysis of the current population (n = 15)

	PFS	95%CI	P	OS	95%CI	P
Surgery						
Yes	6.13	0.70–11.56	0.491	6.13	1.04–11.22	0.605
No	8.83	2.10–15.57		8.83	4.13–13.52	
Gender						
Male	5.43	0–12.83	0.849	5.43	5.11–5.75	0.962
Female	6.23	1.23–11.23		8.83	3.89–13.77	
Apatinib’s dose						
500 mg	8.83	3.03–14.63	0.303	8.83	3.03–14.63	0.448
<500 mg	5.43	2.67–8.19		5.43	3.65–7.21	
The numbers of metastases						
≤2	8.83	3.58–14.08	0.777	4.23	0–10.21	0.966
>2	6.13	4.63–7.63		6.13	4.63–7.63	
Previous treatment						
Second-line	2.50	0–5.57	0.392	5.30	1.45–9.15	0.629
Beyond second-line	6.13	2.05–10.21		6.13	2.05–10.21	

Several studies have shown that S-1 was potentially efficacious for advanced ESCC. Fang et al. [17] have used paclitaxel combined with cisplatin (TP) and S-1 combined with cisplatin (CS) in the unresectable advanced ESCC. The results showed that these therapeutic regimens both achieved satisfactory survival results and the CS group was significantly better than the TP group in terms of compliance, hospital stay, and toxicity. In general, targeted drugs have better safety and tolerability profile compared with chemotherapy drugs.

Multiple targeted drugs have been applied in the treatment of ESCC with first-line chemotherapy failure. Previously, a randomized phase III clinical trial of gefitinib has confirmed its efficacy in esophageal cancer [18]. Actually, gefitinib is a selective epidermal growth factor receptor tyrosine kinase inhibitor (EGFR-TKI), however its efficacy is limited to the esophageal cancer with EGFR gene-sensitive mutations. Moreover, Lorenzen et al. have compared the efficacy of cetuximab combined with cisplatin+5-FU (CET-CF) and cisplatin+5-FU (CF) in the treatment of advanced metastatic ESCC [19], but without clear conclusion. Sunitinib targets VEGFR1–3, PDGFRα, PDGFRβ and c-kit [20]. A phase II study evaluated adjuvant sunitinib following chemoradiotherapy and surgery for locally advanced esophageal cancer. In this study, median survival was 26 months. But all patients were poorly tolerated, and these results were not stratified according to histology [12].

Apatinib mesylate is a small molecule anti-angiogenic targeted drug independently developed by Jiangsu HengRui Medicine Co., Ltd., which could selectively inhibit the activity of VEGFR-2 tyrosine kinase. VEGF could bind to its receptor, and thereby potently inhibit tumor angiogenesis to exert its anti-tumor effects. Usually, malignant tumors are rich in blood vessels, which are the basis of tumor growth and metastasis. The blood vessels could provide nutrients to tumor tissues continuously, and also transport tumor cells to other parts of the body. Given that most solid tumors have a rich blood supply, and thus apatinib is theoretically effective for ESCC. Since apatinib was marketed in 2014, it has been proven to be effective in various cancer, such as gastric cancer [21], liver cancer [22], breast cancer [23], lung cancer [24], ovarian cancer [25], and thyroid cancer [26]. In the current study, the application of apatinib in upper digestive tract tumors mainly focused on the treatment of esophageal adenocarcinoma, gastro-oesophageal junction cancer and gastric cancer [21]. At present, although the efficacy of apatinib monotherapy in advanced ESCC has been confirmed in China, its efficacy is still limited. In this study, the most common AEs occurred in patients who received apatinib combined with S-1 was hypertension and myelosuppression. However, most AEs were mild in severity and resolved soon with treatment interruption and symptomatic treatment. Additionally, only a fewer patients experienced grade 3 or 4 toxicity or serious AEs and no patients experienced irreversible toxicity. Notably, apatinib and S-1 drugs are oral medications that can improve patient’s quality of life and treatment adherence.

In conclusion, the combination therapy of apatinib plus S-1 was effective and well tolerated in the treatment of advanced ESCC patients after first-line chemotherapy failure. The combination therapy has the potential to be a potent therapeutic option for advanced ESCC patients after first-line chemotherapy failure. However, due to the relative small sample size, further studies with a larger number of samples are required to verify our findings.
